# Perceived overall injustice and organizational deviance—Mediating effect of anger and moderating effect of moral disengagement

**DOI:** 10.3389/fpsyg.2022.1023724

**Published:** 2022-12-05

**Authors:** Ge Qin, Lihua Zhang

**Affiliations:** School of Labor and Human Resources, Renmin University of China, Beijing, China

**Keywords:** perceived overall injustice, employee anger, organizational deviance, moral disengagement, emotion

## Abstract

**Introduction:**

This study is dedicated to exploring the influence of perceived overall injustice on employee anger and deviant behavior. Based on fairness heuristic theory and cognitive appraisal theory of emotion, a model was developed to investigate the relationship between perceived overall injustice, anger and organizational deviance. Based on social cognitive theory, the moderating role of moral disengagement was proposed.

**Methods:**

The data were collected from three Chinese manufacturing corporations with a total effective sample size of 264. SPSS 26 and Mplus 8.3 were adopted to analyze data. Confirmatory factor analysis, descriptive statistics analysis and correlation results were illustrated. Hierarchical regression was used to test the model.

**Results:**

Statistical results showed that there is a significant positive relationship among perceived overall injustice, anger and organizational deviance. The moderating effect of moral disengagement on the relationship between perceived overall injustice and organizational deviance is significant, while that on the relationship between anger and organizational deviance is insignificant.

**Discussion:**

This study built a model to discuss the emotional and behavioral influences of perceived overall injustice. The findings suggest that individuals feel more anger as the level of perceived overall injustice increases, which thus lead to higher level of organizational deviance. Morally disengaged employees are more likely to engage in organizational deviance after being treated unfairly. However, the moderating effect of moral disengagement on the relationship of anger and organizational deviance was insignificant. The reason might be because anger is an aggressive emotion and individual experiencing anger may lead to impulsive behavior regardless of moral rules. Implications and limitations have been discussed.

## Introduction

Justice is an important moral principle that means correct in organizations, while injustice means improper and incorrect (Loi et al., 2015). Injustice impacts individuals more significantly than justice, as people usually expect the authority or the organization to be fair (Cropanzano et al., 2001; Judge and Colquitt, 2004). Therefore, once injustice occurs, individuals will take actions to maintain justice (Thompson, 2006). Individual perception of injustice refers to the unfair treatment that employees experience in the organization (Adams, 1965). With the development of the equity theory, organizational behavior researchers attached additional remarks on the definition. In organizational studies, perceived organizational injustice means the perception of unfair treatment that occurs to the employee (Kray and Lind, 2002). According to the definition, perceived injustice has two dimensions. One is when the individual perceives the gain is less than the effort, and the other is when the effort is less than the gain. In this study, we consider the first condition because this type of injustice perception is more harmful, putting employees in a relatively inferior position. This kind of injustice implies that individuals are motivated to overcome injustice, as injustice violates ethical assumptions in the organization (Skitka, 2009; Loi et al., 2012).

Studying perceived injustice in the workplace is crucial to organizational behavior literature. It can cause pernicious damage to individuals and negatively impact organizations (Cohen-Charash and Spector, 2001; Qin et al., 2015). In injustice literature, researchers believe that perceived injustice in the organization can induce mental diseases, harming employees’ health (Greenberg, 2006, 2010; Kivimäki et al., 2007). Perceived organizational injustice arouses employees’ feelings of betrayal, which may evoke employees’ punitive behaviors (Seip et al., 2014). Research has shown that being mistreated harms employee performance, which profoundly impacts the organization. For example, when individuals feel unfair in the organization, their willingness to take positive actions will decrease and they may protest to defend their rights (Skitka and Bravo, 2005). Individuals will experience stronger job stress when they are unfairly treated, so they may refuse to cooperate with other members of the organization or even quit (Alexander and Ruderman, 1987; Lind, 2001; Rizvi et al., 2017).

The first purpose of this study is to discuss the negative effect of perceived injustice from an integrated perspective. Current research has discussed how injustice perceptions formed, yet it is a prevalent opinion that the (in)justice perceptions are based on a specific dimension (Qin et al., 2015). A popular division of (in)justice in previous literature includes distributive (in)justice, procedure (in)justice, interpersonal (in)justice, and informational (in)justice (Colquitt, 2001). Although a large body of organizational behavior research has divided this construct into multiple dimensions, evidence shows that individuals tend to be more concerned with their feelings or experience when (in)justice happens rather than its classification (Shapiro, 2001; Rizvi et al., 2017). Researchers argued that classified (in)justice might not accurately express individuals’ (in)justice experiences (Ambrose and Schminke, 2009). It may be difficult for individuals to calculate their gains and losses in the organizations as Adams (1965) proposed (Lind et al., 1998). When employees lack enough information to assess each type of injustice in the organizations, the perception of a particular injustice experience may be supplementary to other types of injustice (Lind et al., 1998). As a result, employees will form an overall perception of injustice based on unfair phenomena or organizational experiences. This overall injustice perception affects their emotions, leading to anger and hostility (Homans, 1961; Folger, 1986; Smith et al., 1994; Kaya et al., 2016). Individuals may adopt a heuristic approach to form an injustice judgment in a context that lacks information transparency. Therefore, in this research, we regard employees’ perception of organizational injustice as an integrated construct and discuss the negative impact of perceived overall injustice on employee emotions and behaviors.

Second, this study proposes a theoretical framework that integrates perceived overall injustice, anger and organizational deviance based on emotional cognitive appraisal theory. Current empirical research emphasizes injustice influence on individual behaviors or subsequent consequences to the organization. Anger is an aggressive emotional state referring to irritation (Litman et al., 2005). As an emotion, anger, related to hypertension, heart attack, and mental diseases, has been a popular topic of health and psychology studies (Spielberger et al., 1995; Williams et al., 2002).

However, the emotional mechanism of injustice and organizational deviance is underexploited and unvalidated. Although current research proposed potential associations between injustice and employee anger, and explored the link between anger and destructive behaviors (for example, Barclay et al., 2005; Saleem and Khan, 2014), few studies have connected this process. Individual perception of injustice is supposed to result in deviance or retaliation, but we lack evidence on how emotion influence this process. Therefore, we focus on the mediating role of anger. Cognitive appraisal theory provides a framework to answer this question. Employees appraise whether the organization is fair or unfair through judgments. These appraisals can trigger emotional changes (Lazarus, 1991). Justice or injustice will influence employee emotions (Cohen-Charash and Mueller, 2007). Anger is a negative emotion that responds to individual motivation to defend one’s rights and is usually related to hostility and aggression (Frijda et al., 1989; Roseman et al., 1996; Seip et al., 2014). Employees may feel angry when they are unfairly treated, and as a result, they may conduct vindictive behaviors (Barclay et al., 2005). Anger can also be a predictor of other harmful behaviors. For instance, Wang et al. (2018) found that anger leads to aggression. Therefore, our second target is to explore the relationship between perceived overall injustice, anger, and organizational deviance. We provide a new model to explain the effects of perceived injustice.

Finally, this study explores the boundary condition of the perceived overall injustice-anger-organizational deviance model. As mentioned before, employees may have different reactions after being unfairly treated in the organization. Moral disengagement brings further insights into a condition that could exacerbate the destructive influences of injustice perceptions. Moral disengagement was proposed based on social cognitive theory by Bandura (1999, 2002), which refers to cognitive strategies whereby individuals try to separate their behaviors from their moral values to avoid self-sanction when the behaviors violate their morality standards. Being morally disengaged suggests a moral agency that isolating from morality standards will allow individuals to rationalize their behaviors, especially the harmful ones (Bandura, 2002). It can be regarded as an individual tendency to preserve self-esteem, which may give rise to the justification of harmful behaviors and mitigation of responsibilities for the damages (Caprara et al., 2006; 2009).

Most moral disengagement studies trace its antecedents and explore their consequences (Hystad et al., 2014). Few researchers have explored how moral disengagement affects individual behavioral decisions as a state or personal trait. In research that investigates the moderating effect of moral disengagement, scholars found that moral disengagement can be a stimulator when destructive behaviors happen (Wang et al., 2018; Wachs et al., 2022). Individuals who perform deviant behaviors lack a sense of guilt or shame (Loi et al., 2015), which implies that morally disengaged individuals are more likely to engage in destructive actions without scruple. Hence, we propose that moral disengagement may be a catalyst for employees who conduct organizational deviance when they experience prejudice or anger. Employees with higher levels of moral disengagement tend to disregard morality standards and engage in deviance. On the contrary, those with lower moral disengagement are inhibited by their moral values from deviance.

In general, this study concentrates on employees’ organizational perceived overall injustice and explores the impact of this perception. In particular, we build a mechanism that discusses the relationship between perceived overall injustice, anger and organizational deviance and how moral disengagement affects this relationship. We aim to provide empirical evidence to overall injustice literature, supplement workplace emotion research and enrich social cognitive studies. We verified the proposed model with a questionnaire survey carried out for about a month.

## Model construction

### Perceived overall injustice and organizational deviance

Organizational injustice is one of the main concerns for employees because it involves organizational respect for individual ego-esteem. Injustice may subvert employee moral principles in the workplace (Loi et al., 2015). Fairness heuristic theory, based on uncertainty management theory, aims to interpret how individuals form a perception of fairness when they lack enough information to make a judgment (Lind and van den Bos, 2002; Qin et al., 2015). The theory provides a new perspective on justice perception by emphasizing the overall judgment of justice. According to Lind (2001), justice judgment helps individuals understand their relative social status and provides social clues from which individuals realize whether they will be deprived or excluded by the social relations. Therefore, people need to form a quick overall impression of fairness in the organization. The impression may derive from whatever justice information is available. Once individuals have their overall justice impression, it will heuristically affect their attitudes and behaviors in the social groups (Lind, 2001).

In the organization, employees do not rationally analyze the sense of injustice from different dimensions (including distributive, informational, procedural and interpersonal injustice) or make decisions accordingly. Instead, they quickly form injustice impressions through inaccurate or insufficient clues. The perception of injustice in specific dimensions will influence others, leading to an overall injustice perception of the authority or the organization. The approach that the employees obtain injustice information in the organization may be affected by the primacy effect or by a determinant event (van den Bos et al., 1997). They may use such an overall perception of injustice as heuristic information to determine their attitudes and behaviors in the workplace (Lind, 2001; Lind et al., 2001).

According to the fairness heuristic theory, a dilemma occurs when employees join an organization. The organization may use and exploit individuals. When perceived returns do not match the efforts, employees feel unfair. Individuals may be offended by the organization, losing dignity and feeling neglected. Since individuals always have to join a group or an organization, they will weigh the advantages and disadvantages and strive for their material or mental needs. Individuals judge whether they will be expelled based on overall injustice perception. When individuals believe that the organization does not exploit or disrespect them, they will consider that the organization is just and fair. In this case, individuals will consciously abide by the rules and standards of the organization and protect organization interests. They may even sacrifice their interests when a conflict of interest happens (Tyler and Lind, 1992).

On the contrary, when employees have injustice perceptions, they will feel bitter and hurt (Rizvi et al., 2017). Under this circumstance, employees may feel exploited and excluded, which leads to egoism and retaliation. In their perception, the organization is unable to accomplish its promises by the employees, which may result in distrust. Injustice will also bring a sense of disrespect to employees. To compensate for the imbalance, individuals may rebel against the managers or other authorities in the organization. Organizational deviance refers to intentional behaviors that violate organizational norms and threaten the organization’s interest, which stems from organizational injustice (Bennett and Robinson, 2000). Therefore, we propose that employees will engage in organizational deviance when they feel they are being treated unfairly in the organization.

*H1*: Perceived overall injustice is positively related to organizational deviance.

### Mediating role of anger

Emotional cognitive appraisal theory was proposed to explain the process of individual emotions generation. According to this theory, individuals will appraise the events they experienced or perceived, which leads to different emotional reactions. The appraisal is the interaction between the events and individual feelings, while emotion is the adaptive response expressing the appraisal (Lazarus and Folkman, 1984; Lazarus, 1991). Usually, the appraisal is a spontaneous process that is not controlled by the individual (Moors, 2017). In certain conditions, individuals will control their appraisal processes when they recognize that the actor of the event is aware of the action possibilities. This perception is named “affordance” in the appraisal process (Gibson, 1986). Emotion psychologists suggest that appraisal will lead to emotions, feelings, action tendencies, and behaviors (Lazarus, 1991; Roseman and Smith, 2001). In the review of cognitive appraisal theory, Moors et al. (2013) elaborated on the generation of emotions. It contains several processes, including an individual’s interaction with the environment, behavioral tendency, physiological response, expression of emotion, and subjective experience.

In brief, individuals will respond to and express their perceptions of an event or their experience through emotions. In the organization, perceived overall injustice may cause individual appraisal, which arouses emotions. The emotions caused by injustice are usually negative, for example, resentment, anger and disappointment (Folger and Konovsky, 1989). Empirical evidence found that injustice triggers negative responses, such as moral anger, disgust, and retaliation against the organization (Andrews and Kacmar, 2001; Dietz et al., 2003; Rupp and Spencer, 2006).

This may be attributed to the diminishment of self-worth that perceived overall injustice brought to individuals. Individuals’ judgment of justice depends on whether their situation is worthy (Folger and Cropanzano, 2001). It may hurt individuals’ self-esteem when they perceive injustice. In this case, they feel angry, sad, and resentful (Hatfield et al., 2008). The appraisal of the event or the experience is a determinant of individual emotions and feelings (Lazarus, 1991; Roseman and Smith, 2001). Perceived overall injustice implies that the organization or the authority violates normative standards in the workplace (Skitka, 2009). Individuals in such unfair positions perceive themselves to be at a disadvantage. The interpretation of the external environment affects individual emotions and cognition (Moors et al., 2013). Consequently, employees may feel being alienated and threatened. As individuals want to maintain a high level of superiority and self-evaluation, perceived injustice will contribute to degradation and low self-esteem (Smith, 2004; Smith and Kim, 2007). It implies that the organization has violated individual interests and dignity (Loi et al., 2015), which may cause anger.

*H2*: Perceived overall injustice is positively related to employee anger.

Emotions are subjective states that can drive individual motivations and influence behaviors (Ashkanasy and Dorris, 2017). Employees expect for justice when they believe the organization is capable of and should take just actions. If the organization disappoints them, they will feel negative emotions. Anger is one of the most common negative emotions in the workplace (Miron-Spektor and Rafaeli, 2009). It is related to dissatisfaction, hostility, and aggression (Fischer and Roseman, 2007). Psychologists believe angry individuals tend to engage in aggressive behaviors (Colasante et al., 2015; Gresham et al., 2016; Wang et al., 2018), punishments, and retaliations (Barclay et al., 2005). Therefore, anger can be a driving force for harmful behaviors in the organization (Thompson, 2006).

The emotional bond unites the relationship between individual cognition and behavior (Weiss et al., 1999; Barclay et al., 2005). Emotions are the psychological consequences of the individual perception of the external environment, which stimulate individual behaviors (Lazarus, 1991). Anger is often associated with injustice (Thompson, 2006). Previous studies suggested that anger may mediate the relationship between perceived injustice and retaliation (Barclay et al., 2005). Therefore, we propose that anger may cause organizational deviance and play a mediating role between perceived overall injustice and organizational deviance.

*H3*: Employee anger is positively related to organizational deviance.

*H4*: Employee anger mediates the relationship between perceived overall injustice and organizational deviance.

### Moderating role of moral disengagement

Although there may be a correlation between employee perceived overall injustice and organizational deviance, not all unfair treatment can contribute to revenge or destructive behaviors in the workplace. For example, some employees may protest against injustice or leave the organization (Rizvi et al., 2017). Hence, it is important to explore factors that may amplify or attenuate the causal relation between perceived overall injustice and its harmful consequences.

We believe that moral disengagement can affect this relation. In social cognitive theory, individual moral standards can guide and induce them to behave and prevent them from negative behaviors (Bandura et al., 1996). Moral standards are the behavioral guidelines. In general, individuals tend to behave in a way that is consistent with their moral standards (Wang et al., 2018). When individuals violate these standards, it will trigger their moral self-sanction (Bandura et al., 1996). Specifically, when they decide to engage in harmful behaviors, they may develop strategies to avoid moral self-sanction (Bandura, 1999). Moral disengagement indicates the process in which individuals allow themselves to engage in and rationalize behaviors inconsistent with their moral standards through moral self-regulation. It is a psychological scheme by which individuals transform destructive behaviors into reasonable solutions and avoid moral self-sanction (Caprara et al., 2014; Wang et al., 2018). Individuals will justify their negative behaviors by reconstructing their cognitions. In particular, they may interpret their harmful actions as socially valuable or morally acceptable (Bandura, 1999). They can also downplay or overlook the harmful nature of their behaviors. For example, they may claim that their behaviors had caused negligible injury to others or the organization (Gutzwiller-Helfenfinger, 2015; Wang et al., 2018). Moreover, these individuals may obfuscate or contort the attribution of their behaviors, transferring or diffusing their responsibilities (Bandura, 1999; Loi et al., 2015).

Overall, scholars propose that moral disengagement can predict individual negative behaviors in the workplace (Bandura et al., 1996; Barsky, 2011; Gutzwiller-Helfenfinger, 2015). Employees can defend their destructive behaviors, such as theft, by moral disengagement (Dilchert et al., 2007). They can distort the consequences of the events by shifting their responsibilities (Detert et al., 2008; Loi et al., 2015). Those adept at justifying unethical behaviors tend to frequently perform these behaviors as they are less likely to be guilty (Samnani et al., 2014). Thus, individuals with a high level of moral disengagement tend to legitimize the negative consequences they caused. They may convince themselves that the aggressive behaviors are appropriate responses to the injustice they have perceived or experienced. In addition, studies found that when employees feel unfair in the workplace, they may be unwilling to follow moral rules or behave ethically (Cropanzano and Stein, 2009). When these individuals have an unjust perception, they can engage in deviant behaviors without moral burdens. On the contrary, individuals with low levels of moral disengagement are less likely to conduct destructive behaviors even when faced with a disadvantaged situation. When these individuals perceive or experience injustice, they may give up taking revenge on the organization or the authority due to moral beliefs and standards.

*H6*: Moral disengagement moderates the relationship between perceived overall injustice and organizational deviance. In the case of perceived overall injustice, employees with higher moral disengagement are more likely to engage in organizational deviance than those with lower moral disengagement.

Similarly, anger research suggested that the consequences of anger may not be adversarial or aggressive. Although most anger research associated this emotion with hostility and violence (Folger and Baron, 1996), some angry individuals may retaliate in a relatively milder way, such as reducing cooperation or decreasing work efficiency and productivity (Jehn, 1995; Allred et al., 1997; Miron-Spektor and Rafaeli, 2009). Moreover, some researchers suggested that anger is related to positive behaviors because these angry individuals hope to change the irritating situation (Fischer and Roseman, 2007; Lindebaum and Geddes, 2016). Therefore, we believe that moral disengagement can affect the relationship between anger and individual behaviors.

**Figure 1:**
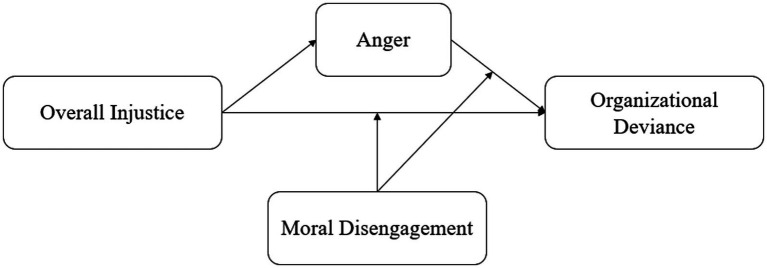
Research model.

In particular, employees with higher moral disengagement view deviant behaviors as a more effective way to deal with anger (Samnani et al., 2014). Employees may argue that deviance is how they vent their anger and blame the organization. Under these circumstances, organizational deviance seems to be reasonable and necessary. By contrast, employees with low moral disengagement tend not to betray their moral rules. These employees regard organizational deviance as incompatible with their principles, having difficulty engaging in disruptive behaviors even though they are angry. Thus, similar to the relationship between perceived overall injustice and organizational deviance, the relationship between anger and organizational deviance may be moderated by moral disengagement. Figure 1 shows the research model.

*H7*: Moral disengagement moderates the relationship between anger and organizational deviance. Angry employees with higher moral disengagement are more likely to engage in organizational deviance than those with lower moral disengagement.

## Procedures and method

### Samples and procedures

We collected data from three manufacturing companies in China. Before the collection, we contacted the leaders of these organizations and obtained their permission to perform the questionnaire survey. The participants are non-managerial employees who work in the departments of finance and accounting, human resource, production, and marketing.

The self-reported questionnaire survey was used to measure the variables. The scales were widely used research scales with good reliability and validity. We adopted translation—back translation to translate all the scales into mandarin and invited friends from different professions to help us know if the scales were ambiguous. All scales were measured on the Likert five-point scale, from 1 (strongly disagree) to 5 (strongly agree). As perceived overall injustice, anger, and moral disengagement involve personal cognition and emotion, these scales should be assessed and reported by the participants. To evaluate organizational deviance, researchers found that the self-reported scale can better reflect and predict employees’ deviant behaviors (Berry et al., 2012). Because the items of most scales that detect deviance include individual behaviors that are difficult for supervisors or colleagues to notice, for example, intentionally reducing work efficiency, disclosure and procrastination (Samnani et al., 2014). Therefore, in this study, all the scales were reported by the participants. To ensure the authenticity of the data, we specifically noted the academic purpose and anonymity at the beginning of each stage, encouraging employees to voice their perceptions and feelings.

Data were collected three times separately, for a total of a month. Each stage of the questionnaires was distributed and collected by the author, with a preset sample size of 338. Consents were collected from all participants before each phase of the survey. We emphasized verbally and in writing that the questionnaire results will not be shared with any third party. The results are entirely confidential and will be only used for academic purposes. At stage 1, the questionnaire included demographic information and the perceived overall injustice scale. Two weeks later, the stage 2 questionnaire included the anger and the moral disengagement scale. Two weeks after stage 2, the stage 3 questionnaire included the organizational deviance scale. To match each round of surveys, we set different numbers at stage 1 and required the interviewees to report their numbers in subsequent surveys. After excluding the questionnaires that could not match, there were 264 valid samples, with an effective rate of 78.11%.

Among the 264 valid samples, 139 were women, accounting for 52.7%. 152 interviewees were men, accounting for 47.3%. The average age was 30.72, ranging from 22 to 49. The average tenure in the current organization was 6.41 years, ranging from 1 year to 25 years. There are four levels of education experiences: high school level (or below), including 12 participants, accounting for 4.5%; college level, including 39 participants, accounting for 14.8%; bachelor’s degree, with 195 participants, accounting for 73.9%; and master’s degree (or above), with 18 participants, accounting for 6.8%.

### Measures

#### Overall perceived injustice

As noted above, perceived injustice includes two explanations. Many researchers have assumed that injustice refers to that individual gains are less than he or she deserves. However, to avoid ambiguity, at the beginning of the overall perceived injustice scale, we emphasized that: “in this questionnaire, ‘injustice’ refers to the situation that one’s gain is beneath his or her effort.” The scale developed by Ambrose and Schminke (2009) was adopted to test this variable. This scale has been used to measure employee perception of overall injustice in the organization with good reliability and validity (Rizvi et al., 2017). It consists of 6 items, including personal assessment and overall perception of organizational injustice. Example items are: “In general, the treatment I receive around here is unfair,” and “Most of the people who work here would say they are often treated unfairly.” α = 0.903.

#### Employee anger

The anger sub-scale from the anger and aggression questionnaire developed by Buss and Perry (1992) was adopted. Interviewees were asked to recall their feelings in the past 2 weeks. There are 7 items, for example, “When frustrated, I let my irritation show,” and “I sometimes feel like a powder keg ready to explode.” α = 0.931.

#### Organizational deviance

The organizational deviance sub-scale from the work deviance scale developed by Bennett and Robinson (2000) was adopted in this study. We asked employees to recall their recent behaviors in the workplace and complete this 12-item scale. Example items are: “I spent too much time fantasizing or daydreaming instead of working,” and “I intentionally worked slower than you could have worked.” α = 0.959.

#### Moral disengagement

The 8-item scale developed by Moore et al. (2012) was used to measure moral disengagement. This scale was based on Bandura’s social cognitive theory, focusing on the test of moral disengagement of employees in the workplace. In this scale, all moral disengagement mechanisms have been included. The scale was widely used to test employee moral disengagement in the workplace (e.g., Beaudoin et al., 2015; Dang et al., 2017). See the literature review by Newman et al. (2020), which applies to our research purpose. Example items are: “people cannot be blamed for doing things that are technically wrong when all their friends are doing it too,” and “people should not be held accountable for doing questionable things when they were just doing what an authority figure told them to do.” α = 0.917.

### Method

We adopted SPSS26 and Mplus8.3 to analyze data. First, we conducted confirmatory factor analysis (CFA) to test the goodness of fit of our model. Second, we examined the descriptive statistics and correlations between each variable. Demographical information was added to the model as the control variable. Hypotheses were tested.

## Results

### Confirmatory factor analysis

Table 1 lists the CFA test. We set five substitutional models to compare with the base model. In Table 1, four-factor model meets the requirements of the goodness of fit, χ^2^ = 988.745, degree of freedom (*df*) = 489, χ^2^/*df* = 2.042, CFI = 0.933, TLI = 0.927, RMSEA = 0.054, SRMR = 0.044. It is significantly superior to other substitutional models. Specifically, single factor model combines all items as one factor, in which χ^2^ = 346.649, *df* = 495, χ^2^/*df* = 7.569, CFI = 0.515, TLI = 0.482, RMSEA = 0.158, SRMR = 0.162. The two-factor model combines overall injustice with anger as one factor and moral disengagement with organizational deviance as one factor, in which χ^2^ = 2849.990, *df* = 494, χ^2^/*df* = 5.770, CFI = 0.648, TLI = 0.624, RMSEA = 0.134, SRMR = 0.168. Three-factor model a combines overall injustice with anger as one factor, in which χ^2^ = 1795.571, *df* = 492, χ^2^/*df* = 3.650, CFI = 0.805, TLI = 0.791, RMSEA = 0.100, SRMR = 0.111. Three-factor model b combines overall injustice with moral disengagement as one factor, in which χ^2^ = 1752.986, *df* = 492, χ^2^/*df* = 3.563, CFI = 0.812, TLI = 0.798, RMSEA = 0.099 SRMR = 0.103. Three-factor model c combines anger with moral disengagement as one factor, in which χ^2^ = 1738.820, *df* = 492, χ^2^/*df* = 3.534, CFI = 0.814, TLI = 0.800, RMSEA = 0.098, SRMR = 0.080.

**Table 1:** Confirmatory factor analysis.

Model	χ^2^	*df*	χ^2^/*df*	CFI	TLI	RMSEA	SRMR
Single factor	3746.649	495	7.569	0.515	0.482	0.158	0.162
2-factor model	2849.990	494	5.770	0.648	0.624	0.134	0.168
3-factor model a	1795.571	492	3.650	0.805	0.791	0.100	0.111
3-factor model b	1752.986	492	3.563	0.812	0.798	0.099	0.103
3-factor model c	1738.820	492	3.534	0.814	0.800	0.098	0.080
4-factor model	988.745	489	2.042	0.925	0.919	0.062	0.044
CMV model	836.380	457	1.830	0.943	0.935	0.056	0.041

We tested common method variance using Harman’s single factor method and adding common method variance factor into the model. The exploratory factor analysis showed that the first factor explained 38.548% of the variance variation, which is less than 40%. Besides, after all items were loaded into the original dimensions, they were simultaneously loaded into an unknown dimension to compare with the four-factor model (Podsakoff et al., 2003). The goodness of fit of the CMV model did not significantly improve, in which χ^2^ = 836.380, *df* = 457, χ^2^/*df* = 1.830, CFI = 0.943, TLI = 0.935, RMSEA = 0.056, SRMR = 0.041. Therefore, the common method variance in this study is acceptable.

### Descriptive statistical analysis

Table 2 shows the means and standard deviation of all variables and the correlation coefficient between each variable. It can be seen that perceived overall injustice is positively correlated with anger (*r* = 0.134, *p* < 0.001) and organizational deviance (r = 0.156, *p* < 0.001). Anger is positively correlated with organizational deviance (r = 0.156, *p* < 0.001). These results initially support our hypothesis.

**Table 2:** Mean, SD, and Correlations.

Variables	Mean	SD	1	2	3	4	5	6	7
1. Gender	0.473	0.499							
2. Age	30.716	5.185	0.176						
3. Education	1.830	0.607	0.009	−0.280					
4. Tenure	6.405	4.237	0.213	15.676***	−0.488**				
5. Perceived overall injustice	3.734	0.592	0.060**	0.248	−0.095***	−0.054			
6. Anger	3.131	0.641	0.026	0.243	−0.056*	0.084	0.134***		
7. Organizational deviance	2.683	0.607	0.032	0.536**	−0.043	0.124	0.179***	0.156***	
8. Moral disengagement	2.723	0.696	−0.034	0.305	−0.119***	0.167	0.156***	0.241***	0.156***

*N* = 264.

*** *p* < 0.001;

** *p* < 0.01;

* *p* < 0.05.

### Hypothesis test

This study used hierarchical regression analysis to test the proposed model. The test results are illustrated in Table 3. First, the main effects (H1, H2, and H3) were examined. In Model 1 and Model 2, anger was the dependent variable to test H1. Model 1 tested the regression results of control variables on employee anger. Model 2 tested the regression of perceived overall injustice on employee anger, β = 0.330 (*p* < 0.001). There is a significant positive correlation between perceived overall injustice and anger, which supports H1. Model 3 and model 4 tested H2. Model 3 tested the relationship between control variables and organizational deviance. In model 4, We added perceived overall injustice into model 3. There was a significant positive correlation between perceived overall injustice and organizational deviance, β = 0.481 (*p* < 0.001). H2 has been supported. In model 5, anger was added to model 3. The correlation between anger and organizational deviance was significantly positive, β = 0.375 (*p* < 0.001), which supports H3.

**Table 3:** Mediation test.

Variables	Anger				Organizational deviance				Organizational deviance			
	Model1		Model2		Model3		Model4		Model5		Model6	
	**β**	SE	**β**	SE	**β**	SE	**β**	SE	**β**	SE	**β**	SE
1 POI			0.330**	0.065			0.481***	0.055			0.396***	0.059
2 Anger									0.375***	0.057	0.255***	0.058
Gender	0.086	0.063	0.020	0.062	0.108	0.061	0.012	0.057	0.079	0.058	0.007	0.055
Age	0.119	0.089	0.053	0.088	0.290**	0.085	0.194*	0.081	0.251**	0.082	0.181*	0.078
Education	−0.154*	0.063	−0.065	0.065	−0.132*	0.062	−0.003	0.059	−0.080	0.059	0.013	0.057
Tenure	−0.092	0.091	−0.014	0.090	−0.195*	0.088	−0.082	0.082	−0.165*	0.083	−0.079	0.079
R^2^	0.035	0.023	0.116**	0.040	0.067*	0.030	0.253***	0.051	0.183***	0.043	0.313***	0.052

This table shows standardized results. *N* = 264. POI = perceived overall injustice.

*** *p* < 0.001;

** *p* < 0.01;

* *p* < 0.05.

Based on Model 2, model 4, and Model 5, we added anger into Model 4 to test the mediating effect of anger. Model 6 tested the mediation result. After adding the mediator, the influence of the independent variable (perceived overall injustice) on the dependent variable (organizational deviance) was significant. Regression coefficient decreased from 0.481 (*p* < 0.001) to 0.396 (*p* < 0.001). Therefore, anger partially mediated the relationship between perceived overall injustice and organizational deviance.

We further used the bootstrap sampling method to verify mediating effect of anger (Hayes, 2013). The indirect effect of anger was 0.090 (*p* < 0.01), SE = 0.032, 95% CI [0.037, 0.161]. The 95% confidence intervals excluded 0, and mediating effect has been verified.

Table 4 illustrates the moderating effect of moral disengagement. Model 7 tested the regression results of control variables on organizational deviance. Model 8 added moral disengagement and perceived overall injustice to model 7. Model 9 added the interaction item of perceived overall injustice and moral disengagement to test the moderating effect of moral disengagement on the relationship between perceived overall injustice and organizational deviance. Results showed that the interaction of perceived overall injustice and moral disengagement has a positive influence on organizational deviance (β = 0.126, *p* < 0.05). This suggests that moral disengagement moderates the relationship between perceived overall injustice and organizational deviance. The higher the moral disengagement, the stronger the relationship between perceived overall injustice and organizational deviance. H5 is supported.

**Table 4:** Moderation test.

Variables	Organizational deviance									
	Model 7		Model8		Model 9		Model 10		Model 11	
	**β**	SE	**β**	SE	**β**	SE	**β**	SE	**β**	SE
OI			0.397***	0.061	0.421***	0.061				
Anger							0.259***	0.071	0.261***	0.071
MD			0.225***	0.062	0.196**	0.065	0.222**	0.073	0.189*	0.079
POI * MD					0.126*	0.063				
Anger * MD									0.082	0.062
Gender	0.108	0.061	0.047	0.056	0.055	0.056	0.106	0.057	0.105	0.058
Age	0.290**	0.085	0.184*	0.078	0.182*	0.077	0.239**	0.08	0.239**	0.081
Education	–0.132*	0.062	0.037	0.058	0.051	0.057	–0.037	0.060	–0.041	0.060
Tenure	–0.195*	0.088	–0.085	0.080	–0.075	0.079	–0.159	0.082	–0.161	0.082
R^2^	0.067*	0.030	0.299***	0.053	0.322***	0.055	0.219***	0.046	0.206***	0.045

*N* = 264. POI = perceived overall injustice, MD = moral disengagement.

*** *p*<0.001;

** *p*<0.01;

* *p*<0.05.

**Figure 2:**
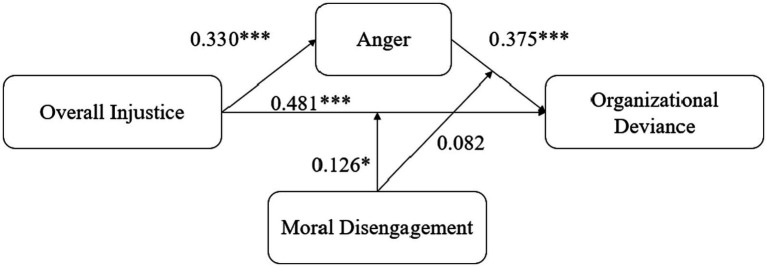
Research results. ***p < 0.001; **p < 0.01; *p < 0.05.

Model 10 and model 11 tested moderating effect of moral disengagement on the relationship between anger and organizational deviance. Model 10 added anger and moral disengagement into model 7. Model 11 added the interaction item of anger and moral disengagement to model 10, which tests the moderating effect of moral disengagement on the relationship between anger and organizational deviance. Results showed that the effect is not significant. H6 is rejected. Figure 2 illustrates the results of the model.

**Figure 3:**
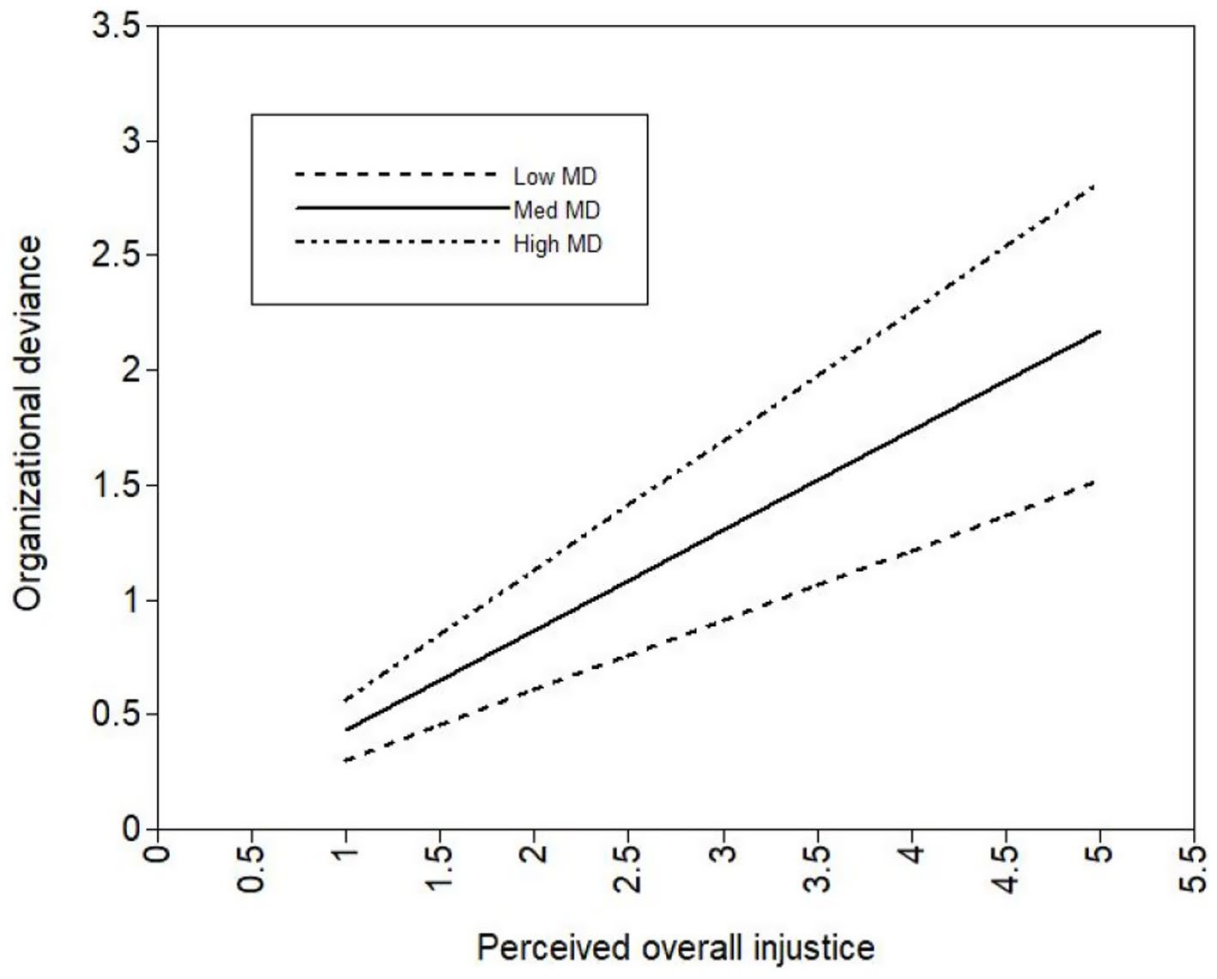
Moderating effect of moral disengagement on the relationship between perceived overall injustice and organizational deviance.

To further examine moderating effect of moral disengagement, we plot the moderating effect of moral disengagement by ±1 standard deviation from the mean of the moderator (Aiken and West, 1991). When the level of moral disengagement is low, the relationship between perceived overall injustice and organizational deviance is positively significant, β = 0.304 (*p* < 0.01, SE = 0.113, bootstrap = 1,000). When the level of moral disengagement is high, the relationship between perceived overall injustice and organizational deviance is positively significant, β = 0.564 (*p* < 0.001, SE = 0.125, bootstrap = 1,000). Figure 3 illustrates the moderating effect of high and low MD. In Figure 3, moral disengagement significantly moderates the relationship between perceived overall injustice and organizational deviance. With the increase in overall injustice, individuals with a higher level of moral disengagement are more likely to conduct deviant behaviors than those with a lower level of moral disengagement. Specifically, when employees have a low overall injustice perception, there is a small gap between deviant behaviors from low and high moral disengagement. When the perception of overall injustice rises, employees with high moral disengagement are more likely to engage in organizational deviance than those with low moral disengagement.

## Discussion

Discussing how overall injustice perception influences individual emotion and behavior is critical to injustice literature. However, although previous studies tested the impacts of injustice perception on individual behaviors in the organization, they emphasize the damage caused by each dimension of injustice (e.g., Loi et al., 2015). In the few studies focusing on the overall perception of injustice, researchers concentrated on work attitudes and behavioral consequences (Barclay et al., 2005). The comparatively scant attention implies that the cognitive emotional process of perceived overall injustice is poorly developed. Therefore, we built a model to discuss the emotional and behavioral influences of perceived overall injustice.

The findings suggest that individuals feel more anger as the level of perceived overall injustice increases, which supports our hypothesis. Consistent with previous studies, perceived overall injustice is positively related to organizational deviance (Barclay et al., 2005). We elucidated the mediating effect of anger through fairness heuristic theory and emotional cognitive appraisal theory. In addition, the process that links perceived overall injustice to organizational deviance is moderated by social cognitive strategy. In our moderation test, morally disengaged employees are more likely to engage in organizational deviance after being treated unfairly. This result follows the ethical characteristics of high moral disengagement individuals who shirk responsibilities and conduct harmful behaviors.

Contrary to our hypothesis, moral disengagement does not affect the relationship between anger and organizational deviance. This could be because anger is an aggressive negative emotion. Individuals who experience anger usually need drastic measures to calm down (Wang et al., 2018). Angry individuals may behave more impulsively and recklessly, which implies that they may not think rationally, and the process may be irrelevant with moral rules. Therefore, the data did not support our hypothesis. Overall, this study supplements the link between perceived overall injustice and destructive behaviors, takes a fresh look at the role of emotion, and sheds light on how moral disengagement, a self-regulating strategy, affects individual behavioral decisions.

## Theoretical and practical implications

Our study makes several important contributions. First, we provide further empirical evidence of the utility of perceived overall injustice to injustice literature. Researchers have long argued that injustice is correlated to destructive behaviors (Aquino and Douglas, 2003; Bobocel, 2013). However, although theorists argue that individuals will utilize every possible injustice information to form judgments (Qin et al., 2015), empirical evidence linking overall injustice and destructive behaviors is still insufficient. Therefore, we dig into an integrated construct of injustice perception, exploring its negative consequences on individual emotion and behavior. Specifically, perceived overall injustice can irritate employees, leading to deviance. We support the fairness heuristic theory, in which the authors proposed that injustice perception can influence individuals as a whole cognition.

We integrated fairness heuristic theory and emotional cognitive appraisal theory to build a model that interprets the cognition-emotion-behavior process of overall injustice. Although the cognitive appraisal process can explain individual cognition issues, scholars who focus on justice or injustice rarely look at this perspective. We find that overall injustice perception has positive relations to anger and organizational deviance, which hence develops cognitive appraisal theory in organizational behavior research. Further, we contribute to anger research by exposing its antecedent and consequence under organizational context. Anger is a common emotion and is supposed to be related to injustice and aggressive behaviors, yet extant findings neglect the integrated link. As discovered by our result, anger can be an expression of employees’ appraisal of unfair perception. The reappraisal of anger may stimulate them to engage in deviant behaviors, which might be due to the desire to appease irritation. We further elucidate the mechanism of employee anger, contributing to workplace emotion study.

Moving beyond the main effects, we also find the moderating role of moral disengagement that exacerbates the relationship between perceived overall injustice and organizational deviance. The results parallel social cognitive theory that suggests individuals adopt regulatory strategy when they perform destructive behaviors that violate their moral standards. Moral disengagement, the crucial cognitive strategy by which individuals get rid of moral self-sanction, explains why deviant behaviors occur when individuals have injustice perceptions. Without the restriction of moral self-sanction, employees considering the organization unfair tend to unload their moral burdens and are more inclined to perform organizational deviance. Whether or not employees are morally disengaged helps explain why some employees choose organizational deviance after being unfairly treated while others do not.

The findings contribute to moral disengagement literature by considering it as a personal state or tendency. In our study, moral disengagement interprets the change in the relationship between perceived overall injustice and organizational deviance. The current discussion about moral disengagement in injustice literature assumes that moral disengagement bridges injustice and harmful behaviors (Hystad et al., 2014; Loi et al., 2015). However, as proposed by social cognitive researchers, moral disengagement may act as an individual tendency or personal trait that enhances the relationship between cognitive process and injurious behavior (Gini et al., 2014, 2015; Wang et al., 2018). We respond to this appeal and explore the regulatory impact of moral disengagement on an individual’s cognition-behavior relation.

Our research has practical implications for organizational management. As general injustice perception may influence employee emotion and behavior, it is not enough to emphasize each dimension of justice. The perception of injustice is uniform and can be affected by different aspects of injustice, such as procedural justice, distributive justice, or interpersonal justice. As injustice is positively related to individual negative emotions and behavior, managers should reduce injustice perceptions as much as possible. Specifically, to alleviate employees’ perception of injustice, managers should pay more attention to any possible situation that may cause injustice. Besides, moral disengagement can accelerate the relationship between perceived overall injustice and organizational deviance. Managers should pay attention to the morality of employees and avoid employees with high level of moral disengagement from performing harmful behaviors in the organization.

## Limitations and future directions

Despite theoretical and practical contributions, this study still has limitations. First, the data were collected from three manufacturing companies in China, which might impact the model generalization. Besides, although we emphasized confidentiality of the survey in each stage of questionnaire collection, this research mainly discusses the variables that may cause negative self-evaluation, such as anger, organizational deviance, and moral disengagement. While answering the questionnaire, the interviewees might avoid or cover up their genuine emotions or tendencies. Moreover, all the questionnaires in this study were self-reported by the employees, leading to inevitable common method deviance. Although statistical tests showed that the common method deviance is acceptable, we recommend future studies to use the other-rated method or other research approaches to further control this problem.

We focus on aggressive negative consequences of perceived overall injustice and anger, a common and popular lens in relevant research. Recently, however, it has been suggested that anger may trigger positive behaviors (e.g., Fischer and Roseman, 2007; Geddes and Callister, 2007; Lindebaum and Geddes, 2016). Future studies can explore what factors may encourage employees to adopt a milder way of solving injustice or anger and how anger may lead to positive impacts. This may help managers better cope with employees’ injustice perception and anger in the workplace.

Our study concentrated on social cognition by discussing the boundary effect of moral disengagement. Moral disengagement is a determinant of individual cognitive and behavioral decisions. However, recent research has found that there might be gender differences in anger and deviant behaviors (e.g., Evers et al., 2011; Björkqvist, 2018). Females seem to less express anger than males and less engage in deviance or retaliation (Chernyak-Hai et al., 2018). This gender difference might be due to physiology (testosterone levels; Archer, 2006) or culture and traditions (Fiske et al., 2002). Therefore, we recommend future research to explore gender differences in anger and organizational deviance.

A final potential direction for future research is to explore the other side of perceived overall injustice. We discussed the condition that the individuals perceive they are deprived of their rights by the organization. The other side of injustice (i.e., the individuals believe they get more than they should) may influence employee emotions and behaviors (Barclay et al., 2005). For instance, when employees unfairly benefit from the organization or colleagues, they may feel guilty and ashamed. Such emotions, different from anger, might lead to various consequences for individuals and organizations. It could cause individuals to compensate the organization (or other employees who suffer losses because of them), for example, by engaging in organizational citizenship behaviors. Further, employees may justify their undeserved benefits. Some employees may try to escape guilt and shame by blaming the unfair results on others’ failures or improving their self-evaluation. Morality might influence this process because individuals with a high level of moral disengagement tend to rationalize the damage they caused. Future research can explore how the other side of overall injustice perception influences employees and organizations.

## Data availability statement

The datasets presented in this article are not readily available because Data were collected for academic purposes only. Requests to access the datasets should be directed to geqin@ruc.edu.cn.

## Author contributions

GQ: conceptualization, methodology, investigation, data analysis, writing original draft. LZ: writing, reviewing and editing. Both authors contributed to the article and approved the submitted version.

## Conflict of interest

The authors declare that the research was conducted in the absence of any commercial or financial relationships that could be construed as a potential conflict of interest.

## Publisher’s note

All claims expressed in this article are solely those of the authors and do not necessarily represent those of their affiliated organizations, or those of the publisher, the editors and the reviewers. Any product that may be evaluated in this article, or claim that may be made by its manufacturer, is not guaranteed or endorsed by the publisher.
